# Understanding Co‐Occurring Comorbidities in Dementia Care: An Exploratory Analysis

**DOI:** 10.1002/alz70858_101689

**Published:** 2025-12-25

**Authors:** Yuchen Zhang, Zhirui Deng, Jennifer B. Seaman, Theresa A. Koleck

**Affiliations:** ^1^ University of Pittsburgh, Pittsburgh, PA, USA

## Abstract

**Background:**

Along with the growing prevalence of Alzheimer's disease and related dementias (ADRD) and the escalating cost of dementia care, people living with dementia (PlwD) experience evolving care needs. These needs stem from the progression of both ADRD and co‐occurring comorbidities, as up to 90% of PlwD are also diagnosed with at least one other chronic, co‐occurring comorbidity. This reality underscores the need for comprehensive dementia care that addresses a wide spectrum of care‐related needs while promoting quality of life in this vulnerable population. Therefore, we aim to characterize and explore the presence and distributions of co‐occurring comorbid conditions in PlwD.

**Method:**

We conducted a cross‐sectional, retrospective exploratory analysis of curated electronic health record data from the *All of Us* Research Program (version 7) during the period of October 1, 2015, to June 30, 2022. Diagnosis of ADRD and comorbidities was defined based on the International Classification of Diseases – Tenth Revision diagnosis codes. We categorized comorbidities based on the Charlson Comorbidity Index. Our sample included *All of Us* participants ≥ 65 years with least one dementia‐related diagnostic code. First, we utilized descriptive statistics and data visualization with UpSet plots for preliminary characterization of patterns of participant comorbidities. Demographic characteristics were compared between participants with and without co‐occurring comorbid conditions using Chi‐square tests or Wilcoxon signed‐rank tests. Then, we conducted a hierarchical clustering analysis using Gower's distance and complete‐linkage clustering, which we visualized with dendrograms.

**Result:**

PlwD (*N* = 4,003) were an average of 73 years of age, 47.5% female, and 72.5% non‐Hispanic White. The most prevalent individual comorbidities included diabetes (67.82%), renal disease (40.24%), chronic pulmonary disease (39.85%), congestive heart failure (37.37%), and peripheral vascular disease (34.57%). While approximately 87% of the PlwD were diagnosed with at least one co‐occurring comorbid condition, we noted heterogenous patterns of comorbidity diagnoses. Our cluster analysis further revealed four distinct comorbidity profiles in PlwD.

**Conclusion:**

Our findings revealed the ubiquitous presence of comorbidities in people living with dementia at a population level. This study marks a crucial first step towards understanding the role of comorbidity profiles in developing person‐centered dementia care.

